# Oxytocin receptor activation does not mediate associative fear deficits in a Williams Syndrome model

**DOI:** 10.1111/gbb.12750

**Published:** 2021-06-10

**Authors:** Kayla R. Nygaard, Raylynn G. Swift, Rebecca M. Glick, Rachael E. Wagner, Susan E. Maloney, Georgianna G. Gould, Joseph D. Dougherty

**Affiliations:** ^1^ Department of Genetics Washington University in St. Louis St. Louis Missouri USA; ^2^ Department of Psychiatry Washington University in St. Louis St. Louis Missouri USA; ^3^ Intellectual and Developmental Disabilities Research Center Washington University in St. Louis St. Louis Missouri USA; ^4^ Department of Cellular and Integrative Physiology University of Texas Health San Antonio San Antonio Texas USA

**Keywords:** associative fear, autoradiography, behavioral genetics, conditioned fear, mouse model, oxytocin, oxytocin receptor antagonist, OXTR, SERT, Williams Syndrome

## Abstract

Williams Syndrome results in distinct behavioral phenotypes, which include learning deficits, anxiety, increased phobias and hypersociability. While the underlying mechanisms driving this subset of phenotypes is unknown, oxytocin (OT) dysregulation is hypothesized to be involved as some studies have shown elevated blood OT and altered OT receptor expression in patients. A “Complete Deletion” (CD) mouse, modeling the hemizygous deletion in Williams Syndrome, recapitulates many of the phenotypes present in humans. These CD mice also exhibit impaired fear responses in the conditioned fear task. Here, we address whether OT dysregulation is responsible for this impaired associative fear memory response. We show direct delivery of an OT receptor antagonist to the central nervous system did not rescue the attenuated contextual or cued fear memory responses in CD mice. Thus, increased OT signaling is not acutely responsible for this phenotype. We also evaluated OT receptor and serotonin transporter availability in regions related to fear learning, memory and sociability using autoradiography in wild type and CD mice. While no differences withstood correction, we identified regions that may warrant further investigation. There was a nonsignificant decrease in OT receptor expression in the lateral septal nucleus and nonsignificant lowered serotonin transporter availability in the striatum and orbitofrontal cortex. Together, these data suggest the fear conditioning anomalies in the Williams Syndrome mouse model are independent of any alterations in the oxytocinergic system caused by deletion of the Williams locus.

## Graphical abstract

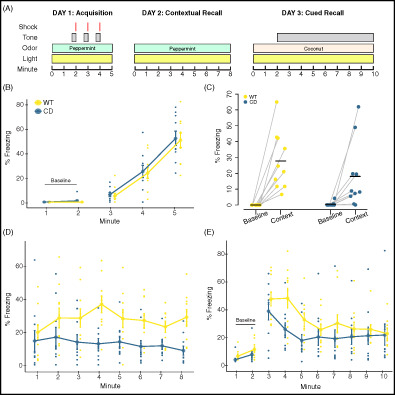

## 1 INTRODUCTION

Williams Syndrome (WS), a multisystemic neurodevelopmental disorder, is caused by a 1.5–1.8 Mbp hemizygous deletion on chromosome 7q11.23, altering the copy number of 26–28 contiguous genes in the WS critical region (WSCR). The complex phenotypic characteristics of WS include craniofacial dysmorphology, connective tissue abnormalities and cardiac problems such as supravalvular aortic stenosis and peripheral artery stenosis. In addition, WS is characterized by distinct cognitive features, including intellectual disability, profoundly impaired visuospatial construction,^1^ atypical facial processing, deficits in motor coordination and control, odynacusis^2^ and impaired auditory processing.^3^, ^4^, ^5^, ^6^, ^7^, ^8^, ^9^, ^10^, ^11^, ^12^ Interestingly, however, individuals with WS possess relatively intact expressive language and verbal skills,^13^, ^14^ as well as heightened sensitivity and emotional response to music.^2^, ^15^ One of the most striking phenotypes of individuals with WS is hypersociability and strong social motivation,^16^, ^17^, ^18^ despite high non‐social anxiety^19^ and deficits in social cognition and awareness.^20^

A substantial body of research indicates the neuropeptide oxytocin (OT) plays a key role in mediating the regulation of social behavior and cognition, fear conditioning and extinction, observational fear,^21^ fear modulation via social memory^22^ and anxiety in humans and rodents.^23^, ^24^, ^25^ Given the aberrant social behavior and anxiety in individuals with WS, recent studies have tested the hypothesis that OT is dysregulated in WS. Indeed, one study found elevated blood levels of OT in individuals with WS compared with controls.^26^ However, the findings on the OT receptor (OXTR) have been contradictory. One study suggested increased gene expression,^27^ while another demonstrated downregulation and hypermethylation of *OXTR* in WS.^28^

While we did not see altered social behavior in a recent application of the standard social approach task, we did see differences in freezing during a conditioned fear task.^29^ Another mouse model, which deletes the entire WS‐homologous region, has also shown alterations in fear conditioning,^30^ and individuals with WS have heightened phobias and non‐social anxieties. Alterations in the brain OT system play an important role in social fear conditioning, contextual fear‐induced freezing and social fear extinction.^31^, ^32^ Additionally, peripheral administration of an OT receptor agonist has been shown to inhibit fear‐induced freezing,^33^ and evoked OT release via channelrhodopsins also results in attenuation of fear.^34^

In this study, we investigated whether OT dysregulation is a mechanism underlying the fear conditioning phenotype following deletion of the WSCR using the mouse experimental system. We employed the model reflecting the most common deletion found in WS patients: the hemizygous loss of the entire genomic region between the *Gtf2i* and *Fkbp6* genes.^30^ These heterozygous Complete Deletion (CD) mice show reduced freezing in fear conditioning recall, which is consistent with the expected consequences of OT elevation. Therefore, we probed whether OT activity could be responsible for the decreased expression of associative fear memory in CD mice. Further, to complement the prior human studies of OXTR expression in peripheral cells,^27^, ^28^ we tested whether OXTR expression differs in CD versus wild type (WT) mice across the brain, but we found no differences after statistical correction in this system, nor in a second neurotransmitter system (serotonin, 5HT), which had previously been shown to cooperate with OT in social learning,^25^ and can be influenced by OT.^35^ Together, these data suggest there is not a direct role for the OT system in associative fear learning in WS.

## 2 MATERIALS & METHODS

### 2.1 Animals

CD mice contain a hemizygous deletion of the WSCR and were maintained on the C57BL/6J background (Jackson #000664).^30^ Animals were bred by crossing CD heterozygotes to C57BL/6J WT animals to produce heterozygous CD experimental mice along with WT littermates for the control group. Tissue collection and genotyping PCR occurred in the second postnatal week. Mice were housed by sex and treatment, when relevant, and were kept on a 12:12 h light/dark schedule with food and water provided ad libitum. All studies were approved by and conducted in accordance with the Institutional Animal Care and Use Committee at Washington University in St. Louis. All behavioral testing occurred during the light phase and was conducted by a female experimenter. Four independent cohorts were used in this study. Cohort 1 included 13 CD and 11 WT male mice from 8 independent litters and was used to assess blood OT levels via ELISA. Cohort 2 comprised 10 CD (4 females (F), 6 males (M)) and 10 WT (8 F, 2 M) mice from four independent litters and were behaviorally examined as adults (postnatal day [P] 97–106) with the conditioned fear task. Cohort 3 comprised 14 CD (8 F, 6 M) and 29 WT (12 F, 17 M) mice from 16 independent litters and served to evaluate the role of the OT system in associative fear and avoidance learning as adults (P68–118). Cohort 4, comprising 22 WT (12 F, 10 M) and 14 CD (5 F, 9 M) from 11 independent litters, was used to evaluate OXTR and serotonin transporter (SERT) expression in specific brain regions of interest. Tissue was collected post‐mortem to confirm initial genotyping results.

### 2.2 Oxytocin ELISA

Blood was drawn from the retro orbital sinus of isoflurane‐anesthetized mice at P30 using heparinized glass capillary tubes. Samples were collected in 1.8 ml EDTA‐coated tubes, spun at 1600 g for 5 min at 4°C, then split into two aliquots, placed on dry ice and stored at −80°C until use. An ELISA kit was used for colorimetric quantification of OT per the manufacturer's protocol (ADI‐900‐153A, Enzo Life Sciences, Farmingdale, NY, USA). Prior to use, samples were diluted 1:3 with 200 μl of assay buffer. Absorbance measurements were read at 405 nm and OT concentration was calculated using a standard curve produced using the provided OT standards.

### 2.3 Conditioned fear task

**FIGURE 1:**
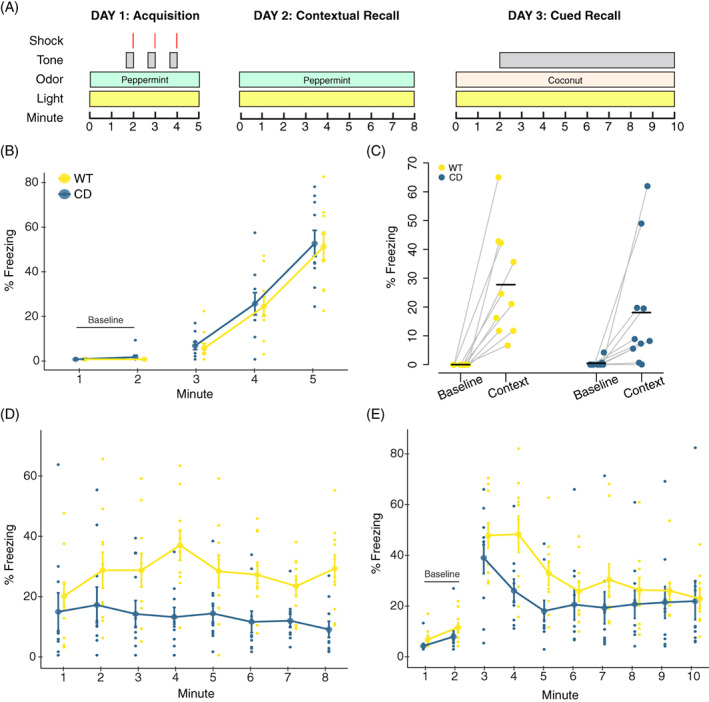
Complete Deletion mice have altered associated fear responses in a conditioned fear task. (A) Overview of the conditioned fear task protocol. (B) Day 1. CD and WT mice show increased freezing with subsequent footshock deliveries. (C) All mice increased freezing in the context associated with the footshock. Baseline is the average of the first 2 min of Day 1. Context is the average of the first 2 min of Day 2. Black bars indicate the mean average percent freezing. Data points of individual mice are connected. (D) Day 2. All mice increased freezing to context relative to Day 1 baseline, although CD mice freeze less than WT mice. (E) Day 3. CD mice have significantly decreased freezing relative to WT mice during minute 4 of tone delivery (*p* = 0.036). WT: *n* = 10; CD: *n* = 10. Connected data points in B, D and E are means ± SEM. Individual scores are represented by colored circles in the background

Associative fear and avoidance learning were evaluated in the CD mice using the conditioned fear paradigm (Figure 1(A)), as described in our previous studies.^36^ Briefly, each mouse was habituated to and tested in an acrylic apparatus, which measured 26 cm × 30 cm × 30.5 cm tall and contained a metal grid floor, an LED light bulb and an inaccessible peppermint odorant, housed within a sound‐attenuating chamber (Actimetrics, Wilmette, IL, USA). The chamber light turned on at the start of each trial and remained illuminated for the duration. On Day 1, the testing session was 5 min. An 80 dB white noise tone sounded for 20 s each at 100 s, 160 s and 220 s. A 1.0 mA shock was paired with the last 2 s of the tone. The baseline freezing behavior (first 2 min) and freezing behavior during the last 2 min was quantified via the computerized image analysis software program FreezeFrame (Actimetrics). This measure allowed for simultaneous visualization of behavior while adjusting a “freezing threshold,” which categorized behavior as freezing or not freezing during 0.75 sec intervals. Freezing was defined as no movement except for normal respiration and data were presented as percent of time spent freezing. Testing on Day 2 lasted 8 min, during which no tones or shocks were presented. This procedure enables evaluation of freezing behavior in response to contextual cues associated with the shock stimulus from Day 1. For the 10 min testing session on Day 3, the context was changed to an opaque Plexiglass‐walled chamber containing a different (coconut) odorant. The 80 dB tone began at 120 s and lasted the remainder of the trial. Freezing during habituation to the new context was quantified across the first 2 min. Freezing behavior to the auditory cue associated with the shock stimulus from Day 1 was quantified for the remaining 8 min. Each day of testing, males were run first, followed by females. Assigned boxes were counterbalanced by genotype. Between runs, the apparatus was cleaned with 70% ethanol (Days 1 and 2) or 0.02% chlorhexidine diacetate solution (Day 3; Zoetis, Parsippany‐Troy Hills, NJ, USA). Animals were put in a holding cage until all cagemates had been tested, then animals were returned to their home cage. Shock sensitivity was evaluated after testing as previously described to verify differences in freezing were not the result of altered sensitivity to the shock stimulus itself.^37^

### 2.4 Intracerebroventricular infusion of oxytocin receptor antagonist during conditioned fear task

The surgical area of adult mice was shaved a day prior to insertion of the guide cannula to facilitate the intracerebroventricular (ICV) injections. Mice were anesthetized with 2.5–5% isoflurane and placed in a stereotaxic apparatus. Prior to the procedure, mice received a local anesthetic, 1 mg/kg of Buprenorphine SR (ZooPharm, Laramie, WY, USA), and an antibiotic, 2.5–5 mg/kg of Baytril (Bayer Healthcare LLC, Shawnee Mission, KS). An incision was made along the skull to visualize bregma to lambda. The periosteum was removed by lightly scratching the surface of the skull and the area was cleaned three times with a betadine solution (Purdue Products L.P., Stamford, CT, USA) on sterile cotton swabs followed by a quick hydrogen peroxide swab. The guide cannula was placed in a stereotaxic cannula holder (#51636–1, Stoelting, Wood Dale, IL, USA). Using a rapid, fluid motion, the 26‐gauge unilateral guide cannula (C315GS‐5/SPC, Plastics One, Roanoke, VA, USA) with dummy cap was inserted at the following coordinates: M/L = +1, A/P = −0.4, D/V = −2.2, based on prior work.^38^, ^39^, ^40^ The guide cannula was cut to a length of 2 mm so that it entered the lateral ventricle. The dummy cap (C315DCS‐5/SPC) and internal cannula (C315IS‐5/SPC) were cut to protrude 0.2 mm from the end of the guide. C&B Metabond dental cement (Parkell, Edgewood, NY, USA) was mixed on a chilled ceramic dish and used to secure the cannula to the skull and seal the surgical area. The dental cement dried completely before the animal was transferred to a recovery cage. Animals were housed together after fully awake and provided 0.25 mg of the chewable anti‐inflammatory Rimadyl (Bio‐Serv, Flemington, NJ, USA). During daily monitoring, dummy caps were replaced and tightened as needed. Mice were euthanized at the first sign of distress or damage to the surgical area and had at least 3 days for recovery prior to testing.

All mice received 1‐μl infusions at least 1 h before each day of the conditioned fear task. Each mouse was given either vehicle (artificial cerebrospinal fluid solution, Tocris Bioscience, Bristol, UK; WT *n* = 16, CD *n* = 7) or an OT receptor antagonist (OTA) (desGly‐NH_2_,d(CH_2_)_5_[Tyr(Me)^2^Thr^4^]OVT, Bachem, Torrance, CA, USA; WT *n* = 13, CD *n* = 7). The OTA, dissolved in vehicle at 1 ng/μl, is a peptidergic ornithine vasotocin analog chosen because of its broad applicability, long half‐life and prior use in ICV injections.^41^, ^42^, ^43^ The solutions were unilaterally delivered into the lateral ventricles through the 33‐gage internal cannula via a PlasticsOne Cannula Connector (C313CS) over the course of 1 min using a Quintessential Stereotaxic Injector (Stoelting #53311) and a 1 μl Hamilton syringe. After injection, 15–30 s passed before removing the internal cannula to ensure proper diffusion. The conditioned fear task was performed as described above. Following completion of behavioral testing, cannula placement was confirmed by injecting enough dye to flood the ventricles and immediately euthanizing the animal via isoflurane overdose. Brains were extracted and sliced coronally at the injection site with a razor blade. Infusion of the dye into the ventricles was then confirmed by eye and samples that missed the ventricles were excluded from the final analysis.

### 2.5 Quantitative autoradiography of OXTR and SERT in mouse brain

Naïve mice were rapidly euthanized by cervical dislocation. Brains were removed, placed in an ice‐cold saline solution for 1 min, then excess saline solution was wicked onto a paper towel. Brains were frozen on crushed dry ice and then stored at −80°C until sliced into 20 μm coronal sections in a cryostat (Leica Biosystems 1850, Buffalo Grove, IL, USA) at −16 to −18°C. Slides were thaw‐mounted onto gelatin‐coated microscope slides, vacuum‐desiccated overnight (18 h) at 4°C, then stored at −80°C until use. Adjacent sections were used for two distinct ligands, [^125^I]OVT and [^125^I]RTI‐55, to assess OXTR and SERT availability, respectively.

#### 2.5.1 OXTR quantitative autoradiography

Binding of iodinated ornithine vasotocin analog ([^125^I]OVT) to OXTR in the mouse brain was performed as described previously,^44^ with minor modifications. Mounted sections were thawed for 30 min at 22–23 °C, then pre‐incubated for 30 min in 50 mM Tris HCl buffer pH 7.4 at 22–23 °C. Next, sections on slides were incubated for 90 min in upright cytomailers filled with 10 ml buffer containing 10 mM MgCl_2_, 0.1% bovine serum albumin and 50 pM [^125^I]OVT (NEX2540, PerkinElmer, Boston, MA, USA). Non‐specific binding was obtained by incubating representative adjacent sections on slides from the series in buffer containing unlabeled OT (1 μM, Ascent Scientific, Bristol, UK). Sections on slides were then washed twice for 5 min each in glass staining dishes containing 300 ml of 4°C buffer and were dipped for 2 s in 4°C deionized water. Slides were dried on a benchtop slide warmer for 1 h or until sections were opaque and any droplets had evaporated.

#### 2.5.2 SERT quantitative autoradiography

Slides were defrosted at 22–23°C for 30 min and pre‐incubated in 30 mM sodium phosphate, 120 mM sodium chloride buffer, pH 7.4 at 22–23°C, for 30 min. For incubation, 100 mM sucrose, 100 nM GBR12909 (to block binding to dopamine transporter) and 50 pM [^125^I]RTI‐55 (NEX272, Perkin Elmer) were added to the buffer. To measure non‐specific binding, 10 μM mazindol was added to a subset of slide mailers containing a representative set of duplicate slides. All unlabeled ligands were from Sigma. Incubation was carried out for 2 h at 22–23 °C. Sections were rinsed twice for 1 min in 4°C buffer (without sucrose), then dipped for 2 s in 4°C deionized water, drained and placed on a slide warmer (Lab‐Line, Fisher Scientific, Pittsburgh, PA, USA), at moderate setting (4 on 10 scale) for 2 h.

#### 2.5.3 Exposure and imaging

Sections on slides were exposed to Biomax MR film (Carestream/Kodak, Rochester, NY, USA) in a cassette for 48 h along with tritium standards (ART0123A, American Radiolabeled Chemicals, St. Louis, MO, USA) calibrated to [^125^I]‐incubated brain mash, as previously described.^45^ Films were developed using an automatic film processor. Digital images of autoradiograms were captured using a 12‐bit CCD monochrome digital camera (CFW‐1612 M, Scion, Frederick, MD, USA) with a 60 mm lens (f‐stop = 4)(Nikon, Melville, NY, USA) mounted on a copy stand (RS‐1, Kaiser Fototechnik, White Plains, NY, USA) with a Kaiser Slimlite Plano LED lightbox. Pixel intensity was calibrated to measure density in units of femtomoles/mg (fmol/mg) protein using a linear function with ImageJ software (https://imagej.nih.gov/ij/download.html).^46^

#### 2.5.4 OXTR data collection

OVT binding to the OXTR was measured in the anterior olfactory nucleus (AON), lateral septal nucleus (LSN), anterior cingulate cortex (ACC), striatum (CPu), hippocampal CA2 and CA3 regions (CA 2/3), paraventricular hypothalamic nucleus (PVN), piriform cortex (Pir), and the combined basolateral and lateral amygdala (BLA & LA) by tracing each region in ImageJ based on coordinates from the Franklin and Paxinos mouse brain atlas.^47^ The AON was traced at Bregma 2.68 mm. The LSN, ACC and CPu were traced between Bregma 0.68 mm and 0.26 mm. The hippocampal CA 2/3 region, PVN and BLA & LA were traced between Bregma −1.58 mm and −1.82 mm. To mitigate potential variability across sections, each region was measured at multiple predetermined locations across consecutive sections within each sample. Nine or more animals were measured per genotype (Table S2).

#### 2.5.5 SERT data collection

SERT availability was measured in the BLA, LA, central amygdala (CeA), ACC, CPu, CA 2/3, insular cortex (IC), lateral parietal association (PtA), nucleus accumbens (NAc), orbitofrontal cortex (OFC), peduncular part of the lateral hypothalamus (PLH) and the bed nucleus of the stria terminalis (BNST). The BLA, LA and CeA were traced between Bregma −1.58 and −1.82 mm. The ACC was traced at Bregma 0.74 mm, the IC was traced at Bregma −1.06 mm and the NAc was traced at 0.74 mm. The CPu was traced between Bregma 0.74 mm and 0.26 mm. The hippocampal CA 2/3 region was traced between Bregma −1.34 mm and −1.58 mm. The PtA and PLH were traced at Bregma −1.58 mm to −1.70 mm. The BNST was traced between Bregma 0.62 mm and −0.22 mm. Up to four independent measurements were taken at the same predetermined locations on consecutive sections within a sample. At least five animals were measured per genotype (Table S2).

### 2.6 Statistical analysis

Analysis for ELISA and the conditioned fear task was completed in R using RStudio (Version 1.2.5019). ELISA analysis utilized the “drc” package.^48^ Conditioned fear data were condensed by minute then assessed for normality, homogeneity of variance and outliers. Data were analyzed with a linear mixed effect model using the “lme4” package,^49^ with genotype as the main factor, and minute as a repeated measure. Tukey's HSD was employed for post hoc analysis. An effect of sex was screened for but not included in the final analysis, as there was no significant effect on any outcome. Detailed outputs for conditioned fear are included in Table S1.

Autoradiography analysis was performed using IBM SPSS Statistics for Windows, Version 26.0. Measurements within a region were averaged to compute the binding value of that region for each sample. These values were normalized by film to compute the total binding value for each region by subtracting the nonspecific binding value of a control sample from the sample binding value. Values less than zero after normalization were treated as zero. Normality, outliers and variance were assessed prior to hypothesis testing. Total OXTR binding values were transformed using a square root transformation to resolve normality violations. A 2 × 2 Analysis of Covariance with fixed factors of sex and genotype and a covariate of age was performed on each region of interest for OXTR autoradiography. For SERT, no transformations were necessary. A 2 × 2 multivariate Analysis of Variance was used to assess factors of sex and genotype across 12 regions of interest. Detailed outputs of statistical tests for all autoradiography experiments are in Table S2.

## 3 RESULTS

### 3.1 Complete deletion mice show impaired contextual and cued fear conditioning

OT has been shown to modulate the freezing response of rodents in conditioned fear tasks. Specifically, central administration of OT results in decreased levels of freezing in response to avoidance‐associated cue or context.^50^ Thus, because of the suggested increase in OT production in WS, we sought to determine whether the CD mouse model also had altered associative fear learning. Previously a decrease in overall freezing time during fear conditioning was shown in the CD model using only male mice;^30^ here we replicate and expand on these findings by confirming the phenotype in both sexes.

We found that CD mice responded to shock during conditioning (Day 1) by increasing freezing to the same extent as WT mice (main effect of minute, *F*(2, 36) = 77.81, *p* = 8.5 × 10^−14^). There was no main or interaction effect of genotype observed (Figure 1(B), complete statistical analysis available in Table S1). WT and CD mice both exhibit significantly higher freezing (*F*(3, 36) = 9.6, *p* = 8.5 × 10^−5^) in the first 2 min of the contextual memory test (context) compared with the first 2 min of training (baseline), indicating each group successfully associated the fear stimuli with the context (Figure 1(C)). On Day 2, CD mice froze less compared with WT mice when placed in the same context (chamber/odor) used for fear conditioning (Figure 1(D), main effect of genotype, *F*(1, 18) = 7.95, *p* = 0.011), indicating impaired contextual associative fear memory. The response to the conditioned cue was also decreased in CD mice but not as broadly, with a main effect of minute (*F*[9162] = 22.0195, *p* = 2 × 10^−16^), and a borderline minute by genotype interaction (Figure 1(E), *F*(9,162) = 1.9151, *p* = 0.053), but no main effect of genotype. Post hoc analysis revealed the mean freezing of CD mice was almost 24% less than WT freezing at minute 4 (*p* = 0.036). Overall, CD mice show a deficit in contextual fear conditioning that is consistent with elevated OT activity.

### 3.2 CD conditioning deficits are not reversed by central infusion of an oxytocin receptor antagonist

**FIGURE 2:**
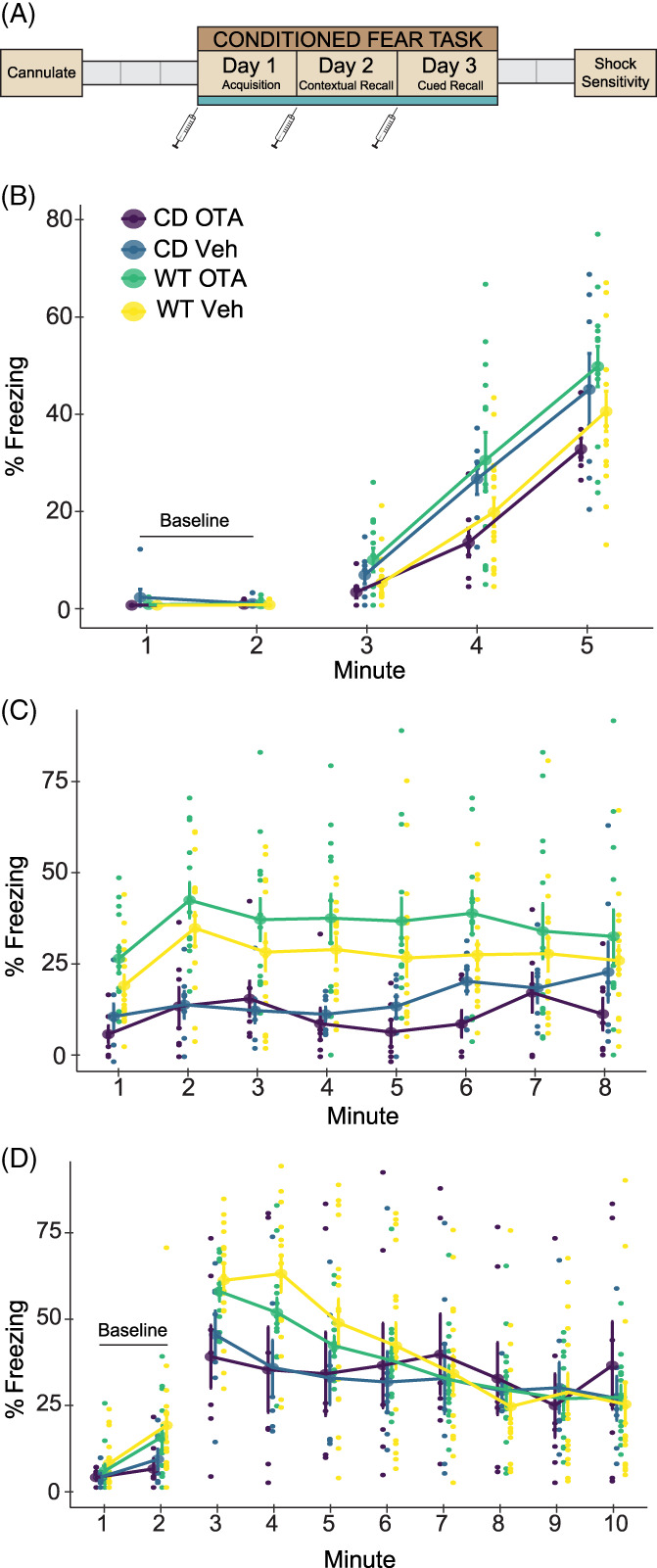
Central administration of an oxytocin receptor antagonist does not rescue reduced contextual or cued fear responses in CD mice. (A) Schematic of the experiment. Gray boxes show rest days. Syringes indicate ICV infusions of OTA or vehicle, which occurred at least 1 hour prior to testing. (B) Day 1 of conditioned fear. CD and WT mice show increased freezing with subsequent footshock deliveries. WT OTA‐treated mice freeze significantly more than CD OTA‐treated mice. (C) Day 2. All mice show increased freezing to context (minutes 1 and 2) relative to Day 1 baseline. CD mice freeze less than WT mice but there is no main or interaction effect of treatment. (D) Day 3. CD mice have significantly decreased freezing relative to WT during minute 4 of tone delivery (*p* = 0.008), but there is no effect of treatment. WT Veh: *n* = 16; WT OTA: *n* = 13; CD Veh: *n* = 7; CD OTA: *n* = 7. Connected data points are means ± SEM. Individual scores are represented by smaller unconnected circles. OTA, oxytocin receptor antagonist; Veh, vehicle

We next sought to determine if CD mice had elevated OT levels in the blood, as had been reported in patients.^26^ We used the same ELISA approach, but did not see a significant difference between genotypes (*t* = 0.003, *df* = 21.664, *p* = 0.9976; CD (*n* = 13): *M* = 1138.191 pg/ml, *SD* = 503.682; WT (*n* = 11) *M* = 1138.791 pg/ml, *SD* = 478.916). However, even WT mice had a remarkable range of OT levels in blood (82–1731 pg/ml), likely driven by the periodic nature of OT release, coupled with its short half‐life (<5 min).^51^ Thus, it can be hard, at a single point in time, to detect and make conclusions about average OT levels. Furthermore, blood OT levels may not reflect OT levels in the brain. Therefore, we took an experimental approach; if elevated central OT was responsible for decreased associative fear learning in CD mice, then blocking central OXTR activity should reverse the phenotype. We implanted CD and WT mice with ICV cannulas to directly administer an OXTR peptide antagonist (OTA) and block all receptor activity during the fear conditioning procedure (Figure 2(A)).

There were no baseline freezing differences between CD and WT animals, as measured in minutes 1 and 2 of the first day of testing (*F*(1, 39)=1.58, *p* = 0.22). During the conditioning phase, we found an interaction of genotype and treatment. The OTA had an opposite effect on freezing in CD and WT animals. Specifically, WT mice receiving the OTA froze more than their CD counterparts (Figure 2(B), *F*(1, 39) = 7.32, *p* = 0.0101).

During contextual fear recall, both CD and WT animals showed evidence of learning, as freezing increased in all groups from Day 1 baseline compared with the first 2 min of Day 2. Overall, a main effect of genotype on contextual fear memory (Figure 2(C) *F*(1, 39) = 19.96, *p* < 6.6 × 10^−5^) reflects a significant reduction in freezing within CD mice compared with WT mice, regardless of treatment. While this replicated results from our first experiment, there was no main effect of treatment or a genotype by treatment interaction, thus administration of the OTA did not significantly alter contextual fear responses. This was also true on Day 3 for cued fear responses (Figure 2(D)), where there was only a main of effect of time (*F*[2273] = 26.45, *p* = 2.2 × 10^−16^) and an interaction between time and genotype (*F*[2273] = 5.88, *p* = 2.26 × 10^−6^) driven by the decreased freezing of CD mice compared with WT mice at minute 4 (*p* = 0.0083). Thus, we show OT signaling at the OXTR does not account for the impaired associative fear response in this model.

### 3.3 Autoradiography reveals no changes in oxytocin receptor density or distribution in CD mice

**FIGURE 3:**
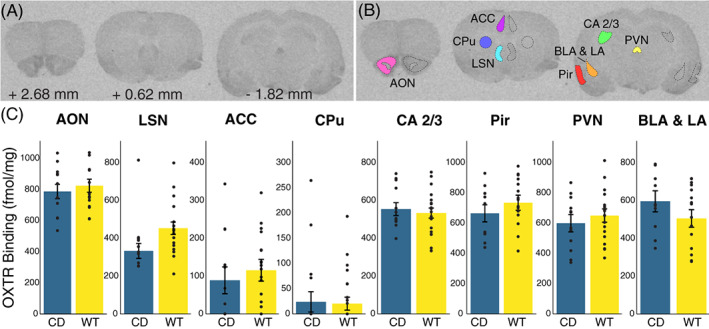
No significant differences in oxytocin receptor density in Complete Deletion mice. (A) Coronal sections from a representative mouse brain used in iodinated ornithine vasotocin analog ([^125^I]OVT) autoradiography analysis with corresponding distance from bregma. (B) Example tracing of regions of interest including anterior olfactory nucleus (AON), cingulate cortical areas 1 and 2 (ACC), striatum (CPu), lateral septal nucleus (LSN), hippocampal CA 2 and 3 regions, paraventricular nucleus (PVN), basolateral (BLA) and lateral (LA) amygdala, and piriform cortex (Pir). (C) Results of oxytocin receptor autoradiography comparing [^125^I]OVT binding (fmol/mg of protein) in regions of interest between CD mice and WT mice. Colored bars show means ± SEM (brackets). Individual averaged measurements for each mouse are represented by circles. For each genotype, *n* ≥ 9

In parallel to the experimental approach, we investigated OXTR availability in the mouse brain through a discovery‐based approach, as elevated levels in the amygdala might influence fear conditioning.^50^ Particularly, given opposite directions of effect of the OTA in WT and CD mice on Day 1 freezing (Figure 2(B)), we suspected genotype differences in OXTR expression in regions related to fear learning. Furthermore, it is of interest to study this binding given the findings of OXTR dysregulation in brains of humans with the WSCR deletion.^27^, ^28^ Therefore, we conducted an autoradiography study using radiolabeled OVT ligand on coronal sections of WT and CD brains (Figure 3(A)). As well as measuring regions relevant to fear conditioning (BLA and LA^52^, ^53^; LSN^31^), we measured areas of where OT has been shown to affect sociability or memory (AON^54^; LSN^55^; ACC^56^, ^57^; CA2/3^58^, ^59^; Pir^60^; PVN^61^; and CPu^25^; Figure 3(B)), as hypersociability and cognitive impairments are characteristic of WS. We found no significant differences in OXTR binding between genotypes within regions of interest in CD and WT brains when corrected for multiple testing (Figure 3(C), Supplemental Table 2). There was a nominally significant change in the LSN (*p* = 0.034), but it did not meet the corrected experimentwise critical alpha level (α = 0.006). Given the role of the LSN in fear and anxiety and future focused studies of this region may be warranted.

### 3.4 Autoradiography reveals no changes in serotonin transporter density or distribution in CD mice

Finally, with no changes in OXTR availability, we examined an additional alternative neurotransmitter: serotonin (5HT). Disruption to the 5HT system in WS has been suggested in prior studies. Specifically, Proulx et al. (2010) determined that there are enhanced 5‐HT_1A_ receptor‐mediated currents in a WS mouse model with low innate anxiety.^62^ More recently, Lew et al. (2020) compared serotonergic innervation in the amygdala between autism and WS in human postmortem samples, concluding that there is decreased innervation in WS brains compared with neurotypical brains.^63^ Therefore, we focused on the SERT, as it should provide a measure of serotonergic innervation to different structures.

**FIGURE 4:**
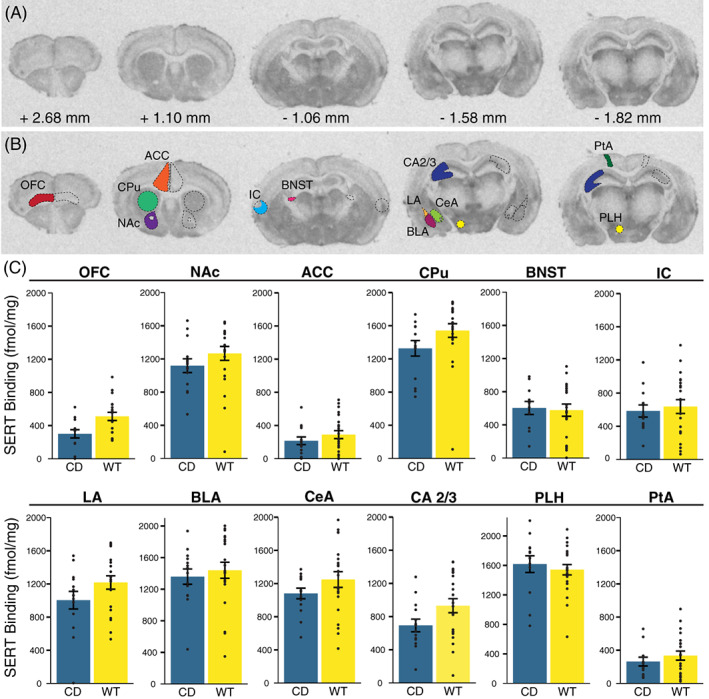
No significant differences in serotonin transporter density in Complete Deletion mice. (A) Coronal sections with corresponding distance from bregma from a representative mouse brain used in [^125^I]RTI‐55 autoradiography analysis. (B) Example tracing of regions of interest including orbitofrontal cortex (OFC), anterior cingulate cortex (ACC), striatum (CPu), nucleus accumbens (NAc), the bed nucleus of the stria terminalis (BNST), insular cortex (IC), hippocampal CA 2 and 3 (CA 2/3) regions, basolateral (BLA), lateral (LA), and central (CeA) amygdala, lateral parietal association (PtA) and the peduncular part of the lateral hypothalamus (PLH). (C) Results of serotonin transporter (SERT) autoradiography comparing [^125^I]RTI‐55 binding (fmol/mg protein) in regions of interest between CD mice and WT mice. Colored bars show means ± SEM (brackets). Individual measurements for each mouse (regional average) are represented by circles. For each genotype, *n* ≥ 5

We measured SERT binding in several brain regions (Figure 4(A)). We focused on amygdalar regions relevant to the human postmortem studies^63^ and fear conditioning phenotypes,^64^ assessing BLA, central amygdala (CeA) and LA regions independently based on findings that they differ in the amount of serotonergic innervation in some species.^65^ We then included other areas where SERT has been implicated in behaviors relevant to the WS phenotype, patient findings and knowledge of 5HT biology. These included the nucleus accumbens (NAc) based on 5HT's role on social reward in this region,^25^ the BNST for its role in adaptive anxiety,^66^ and additional hypothalamic and cortical regions of interest, including the ACC, lateral parietal association (PtA), orbitofrontal cortex (OFC) and the peduncular part of the lateral hypothalamus (PLH) which is where the medial forebrain bundle is found, representing a major ascending pathway for nearly all 5HT axons (Figure 4(B)).^67^ Overall, there was a nonsignificant cross‐region effect of genotype (*F*(12) = 2.41; *p* = 0.07), with no effect of sex (*F*(12) = 0.49; *p* = 0.89) (Figure 4(C)). Nonsignificant decreases in SERT density in CD mice compared with WT were found in the CPu (*p* = 0.067) and OFC (*p* = 0.060), and these may be potential regions of interest for future, focused studies better powered to study smaller effects.

## 4 DISCUSSION

We and others have previously observed altered fear conditioning in WS models.^29^, ^30^ Expanding on previous studies that used only male mice, we found, using both sexes, that CD animals on a C57BL/6J background had a suppressed fear response to the context and cue presented in the fear conditioning paradigm. Thus, CD mice enable investigation of the underlying circuit disruptions mediating this phenotype. We hypothesized that the altered associative fear memory response in CD mice was because of the increased availability of OT based on human findings of elevated OT in WS.^26^ We focused our efforts on functional studies, which would be definitive with regards to a role for OT in fear conditioning of CD mice. Using an intraventricular cannula, we treated mice with an OT receptor antagonist during each day of conditioned fear to attempt to counteract any possible effect of increased OT production on the fear response. The OTA did not alter the subdued freezing response in CD mice. Therefore, the associative fear conditioning phenotype that results from loss of the WS critical region is not mediated by OXTR activity.

Our results tell us less about the role of OT in WT mice. While many prior studies show OT modulating fear conditioning,^33^, ^34^, ^50^, ^53^, ^68^ Pisansky et al. (2017) found OT enhances fear in a social paradigm of observational fear learning, but does not affect non‐social fear learning.^21^ We did not see a significant difference in WT mouse behavior in response to OTA (*p* < 0.083, Day 2), but as there was a small difference in the means of WT mice given Vehicle compared with those given OTA, there may be an effect below our power to detect. Therefore, we are hesitant to weigh in on this debate. In the end, the discrepancy across studies could be a result of dosage. Gunduz‐Cinar et al. (2020) show different concentrations of OT have opposing effects on other fear‐related tasks.^69^ We have also observed an effect of genetic background on fear conditioning in the CD mice, with elevated freezing in CD mutants on an FVB/AntJ x C57BL/6J F1 hybrid background.^29^ These hybrids show different levels of baseline freezing in response to cues even in WT animals, suggesting the impact of the CD deletion interacts with other genes in the genome to modulate conditioned fear effects. Thus, if OT is not involved, a genetic screen for interacting loci using mouse strain panels may help identify the relevant pathways. Another option would be to investigate genes based on dysregulated expression in models of WS, such as the serotonin 5HT_1B_ receptor, which is among the top 10 dysregulated genes in a cell model of WS.^70^

In addition, given some of the prior work suggesting OXTR receptor gene expression and methylation in WS patient blood cells, we were curious if receptor availability was also altered in the brain following deletion of these genes. We used autoradiography because it can measure the availability of the receptor at the surface, which should reflect protein level and localization changes in addition to changes in gene expression. Further, it provides an opportunity for spatially informed analyses. As such, it is the best single measure for assessing if the OXTR is modulated in a conserved way by these mutations. We assessed regions previously associated with fear conditioning and those where OT had been shown to modulate social reward (CPu). Overall, we found no significant changes in OXTR binding in the CD mouse brain compared with controls, although there was a nominally significant difference in the LSN prior to correction, in the same direction of effect as seen in WS patient blood cells. The LSN is an interesting center integrating a variety of fear and anxiety signals, for example, playing a role in how stressful social cues are received.^71^ In addition, the LSN has been associated with fear‐enhancing effects of the OXTR, but as modulation of the OXTR did not alter contextual conditioned fear responses, it is thought to be through indirect means.^72^ These data motivate future studies focusing on the role of the LSN in behavioral abnormalities observed in the CD mouse model.

Despite ruling out a direct role for OT in fear conditioning deficits in this model, altered OT might play a role in the increased social motivation in this population. While it is of interest scientifically to assess this, hypersociality is not as much of a concern therapeutically as other phenotypes, such as learning deficits, ADHD, phobias and anxiety. Beyond OT and serotonin, dopamine has also been implicated in WS,^7^ and has been previously connected to fear conditioning,^73^ other anxiety‐avoidance tasks,^74^ and ADHD‐related hyperactivity.^75^ Thus, it is possible deletion of the WSCR disrupts dopamine signaling to result in these behavioral alterations. Understanding the roles of any of these systems in patient‐related phenotypes of the CD mice might help highlight potential treatments in WS, as a wide range of therapeutics working on these systems are currently available.

## Supporting information


**TABLE S1** Supplementary Table 1

**TABLE S2** Supplementary Table 2

## Data Availability

The data that support the findings of this study are available from the corresponding author upon reasonable request.

## References

[1] Mervis CB , Robinson BF , Pani JR . Visuo‐spatial construction. Am J Hum Genet. 1999;65(5):1222‐1229. 10521286 10.1086/302633 PMC1288273

[2] Levitin DJ , Cole K , Lincoln A , Bellugi U . Aversion, awareness, and attraction: investigating claims of hyperacusis in the Williams Syndrome phenotype. J Child Psychol Psychiatry. 2005;46(5):514‐523. 10.1111/j.1469-7610.2004.00376.x. 15845131

[3] Morris CA , Demsey SA , Leonard CO , Dilts C , Blackburn BL . Natural history of Williams Syndrome: physical characteristics. J Pediatr. 1988;113(2):318‐326. 10.1016/S0022-3476(88)80272-5. 2456379

[4] Dilts CV , Morris CA , Leonard CO . Hypothesis for development of a behavioral phenotype in Williams Syndrome. Am J Med Genet. 1990;37(S6):126‐131. 10.1002/ajmg.1320370622. 2118772

[5] Donnai D , Karmiloff‐Smith A . Williams Syndrome: from genotype through to the cognitive phenotype. Am J Med Genet. 2000;97(2):164‐171. 10.1002/1096-8628(200022)97:2<164::AID-AJMG8>3.0.CO;2-F. 11180224

[6] Mervis CB , Robinson BF , Bertrand J , Morris CA , Klein‐Tasman BP , Armstrong SC . The Williams Syndrome cognitive profile. Brain Cogn. 2000;44(3):604‐628. 10.1006/brcg.2000.1232. 11104544

[7] Gagliardi C , Martelli S , Burt MD , Borgatti R . Evolution of neurologic features in Williams Syndrome. Pediatr Neurol. 2007;36(5):301‐306. 10.1016/j.pediatrneurol.2007.01.001. 17509461

[8] Martens MA , Wilson SJ , Reutens DC . Research review: Williams Syndrome: a critical review of the cognitive, behavioral, and neuroanatomical phenotype. J Child Psychol Psychiatry. 2008;49(6):576‐608. 10.1111/j.1469-7610.2008.01887.x. 18489677

[9] Doherty‐Sneddon G , Riby DM , Calderwood L , Ainsworth L . Stuck on you: face‐to‐face arousal and gaze aversion in Williams Syndrome. Cogn Neuropsychiatry. 2009;14(6):510‐523. 10.1080/13546800903043336. 19736593

[10] Riby D , Hancock PJB . Looking at movies and cartoons: eye‐tracking evidence from Williams Syndrome and autism. J Intellect Disabil Res. 2009;53(2):169‐181. 10.1111/j.1365-2788.2008.01142.x. 19192099

[11] Morris CA . The behavioral phenotype of Williams Syndrome: a recognizable pattern of neurodevelopment. Am J Med Genet C Semin Med Genet. 2010;154C(4):427‐431. 10.1002/ajmg.c.30286. 20981771

[12] Barak B , Feng G . Neurobiology of social behavior abnormalities in autism and Williams Syndrome. Nat Neurosci. 2016;19(6):647‐655. 10.1038/nn.4276. 29323671 PMC4896837

[13] Bellugi U , Lichtenberger L , Jones W , Lai Z , St. George M . I. The neurocognitive profile of Williams Syndrome: a complex pattern of strengths and weaknesses. J Cogn Neurosci. 2000;12(suppl. 1):7‐29. 10.1162/089892900561959. 10953231

[14] Fishman I , Yam A , Bellugi U , Mills D . Language and sociability: insights from Williams Syndrome. J Neurodev Disord. 2011;3(3):185‐192. 10.1007/s11689-011-9086-3. 21671048 PMC3261273

[15] Levitin DJ , Menon V , Schmitt JE , et al. Neural correlates of auditory perception in Williams Syndrome: an fMRI study. Neuroimage. 2003;18(1):74‐82. 10.1006/nimg.2002.1297. 12507445

[16] Korenberg JR , Chen X‐N , Hirota H , et al. VI. Genome structure and cognitive map of Williams Syndrome. J Cogn Neurosci. 2000;12(suppl 1):89‐107. 10.1162/089892900562002. 10953236

[17] Meyer‐Lindenberg A , Mervis CB , Berman KF . Neural mechanisms in Williams Syndrome: a unique window to genetic influences on cognition and behaviour. Nat Rev Neurosci. 2006;7(5):380‐393. 10.1038/nrn1906. 16760918

[18] Järvinen A , Korenberg JR , Bellugi U . The social phenotype of Williams Syndrome. Curr Opin Neurobiol. 2013;23(3):414‐422. 10.1016/j.conb.2012.12.006. 23332975 PMC4326252

[19] Ng‐Cordell E , Hanley M , Kelly A , Riby DM . Anxiety in Williams Syndrome: the role of social behaviour, executive functions and change over time. J Autism Dev Disord. 2018;48(3):796‐808. 10.1007/s10803-017-3357-0. 29124472 PMC5847160

[20] Klein‐Tasman BP , Li‐Barber KT , Magargee ET . Honing in on the social phenotype in Williams Syndrome using multiple measures and multiple raters. J Autism Dev Disord. 2011;41(3):341‐351. 10.1007/s10803-010-1060-5. 20614173 PMC3020248

[21] Pisansky MT , Hanson LR , Gottesman II , Gewirtz JC . Oxytocin enhances observational fear in mice. Nat Commun. 2017;8(1):2102. 10.1038/s41467-017-02279-5. 29235461 PMC5727393

[22] Guzmán YF , Tronson NC , Sato K , et al. Role of oxytocin receptors in modulation of fear by social memory. Psychopharmacology (Berl). 2014;231(10):2097‐2105. 10.1007/s00213-013-3356-6. 24287604 PMC4004649

[23] Heinrichs M , von Dawans B , Domes G . Oxytocin, vasopressin, and human social behavior. Front Neuroendocrinol. 2009;30(4):548‐557. 10.1016/j.yfrne.2009.05.005. 19505497

[24] Meyer‐Lindenberg A , Domes G , Kirsch P , Heinrichs M . Oxytocin and vasopressin in the human brain: social neuropeptides for translational medicine. Nat Rev Neurosci. 2011;12(9):524‐538. 10.1038/nrn3044. 21852800

[25] Dölen G , Darvishzadeh A , Huang KW , Malenka RC . Social reward requires coordinated activity of nucleus accumbens oxytocin and serotonin. Nature. 2013;501(7466):179‐184. 10.1038/nature12518. 24025838 PMC4091761

[26] Dai L , Carter CS , Ying J , Bellugi U , Pournajafi‐Nazarloo H , Korenberg JR . Oxytocin and vasopressin are Dysregulated in Williams Syndrome, a genetic disorder affecting social behavior. Plos One. 2012;7(6):e38513. 10.1371/journal.pone.0038513. 22719898 PMC3373592

[27] Haas BW , Smith AK . Oxytocin, vasopressin, and Williams Syndrome: epigenetic effects on abnormal social behavior. Front Genet. 2015;6(28). 10.3389/fgene.2015.00028. PMC4330921 25741359

[28] Kimura R , Tomiwa K , Inoue R , et al. Dysregulation of the oxytocin receptor gene in Williams Syndrome. Psychoneuroendocrinology. 2020;115:104631. 10.1016/j.psyneuen.2020.104631. 32114409

[29] Kopp N , McCullough K , Maloney SE , Dougherty JD . Gtf2i and Gtf2ird1 mutation do not account for the full phenotypic effect of the Williams Syndrome critical region in mouse models. Hum Mol Genet. 2019;28(20):3443‐3465. 10.1093/hmg/ddz176. 31418010 PMC7343053

[30] Segura‐Puimedon M , Sahún I , Velot E , et al. Heterozygous deletion of the Williams‐Beuren Syndrome critical interval in mice recapitulates most features of the human disorder. Hum Mol Genet. 2014;23(24):6481‐6494. 10.1093/hmg/ddu368. 25027326

[31] Zoicas I , Slattery DA , Neumann ID . Brain oxytocin in social fear conditioning and its extinction: involvement of the lateral septum. Neuropsychopharmacology. 2014;39(13):3027‐3035. 10.1038/npp.2014.156. 24964815 PMC4229574

[32] Hasan MT , Althammer F , Silva da Gouveia M , et al. A fear memory engram and its plasticity in the hypothalamic oxytocin system. Neuron. 2019;103(1):133‐146.e8. 10.1016/j.neuron.2019.04.029 31104950

[33] Modi ME , Majchrzak MJ , Fonseca KR , et al. Peripheral administration of a long‐acting peptide oxytocin receptor agonist inhibits fear‐induced freezing. J Pharmacol Exp Ther. 2016;358(2):164‐172. 10.1124/jpet.116.232702. 27217590 PMC4959095

[34] Knobloch HS , Charlet A , Hoffmann LC , et al. Evoked axonal oxytocin release in the central amygdala attenuates fear response. Neuron. 2012;73(3):553‐566. 10.1016/j.neuron.2011.11.030. 22325206

[35] Mottolese R , Redouté J , Costes N , Le Bars D , Sirigu A . Switching brain serotonin with oxytocin. Proc Natl Acad Sci U S A. 2014;111(23):8637‐8642. 10.1073/pnas.1319810111. 24912179 PMC4060712

[36] Maloney SE , Rieger MA , Al‐Hasani R , Bruchas MR , Wozniak DF , Dougherty JD . Loss of CELF6 RNA binding protein impairs cocaine conditioned place preference and contextual fear conditioning. Genes Brain Behav. 2019;18(7):e12593. 10.1111/gbb.12593. 31215739 PMC7059558

[37] Khuchua Z , Wozniak DF , Bardgett ME , et al. Deletion of the N‐terminus of murine map2 by gene targeting disrupts hippocampal ca1 neuron architecture and alters contextual memory. Neuroscience. 2003;119(1):101‐111. 10.1016/s0306-4522(03)00094-0. 12763072

[38] Raposinho PD , Pierroz DD , Broqua P , White RB , Pedrazzini T , Aubert ML . Chronic administration of neuropeptide Y into the lateral ventricle of C57BL/6J male mice produces an obesity syndrome including hyperphagia, hyperleptinemia, insulin resistance, and hypogonadism. Mol Cell Endocrinol. 2001;185(1):195‐204. 10.1016/S0303-7207(01)00620-7. 11738809

[39] Radulovic J , Rühmann A , Liepold T , Spiess J . Modulation of learning and anxiety by corticotropin‐releasing factor (CRF) and stress: differential roles of CRF receptors 1 and 2. J Neurosci. 1999;19(12):5016‐5025. 10366634 10.1523/JNEUROSCI.19-12-05016.1999 PMC6782638

[40] Mantella RC , Vollmer RR , Li X , Amico JA . Female oxytocin‐deficient mice display enhanced anxiety‐related behavior. Endocrinology. 2003;144(6):2291‐2296. 10.1210/en.2002-0197. 12746288

[41] Ferguson JN , Young LJ , Hearn EF , Matzuk MM , Insel TR , Winslow JT . Social amnesia in mice lacking the oxytocin gene. Nat Genet. 2000;25(3):284‐288. 10.1038/77040. 10888874

[42] Ferguson JN , Aldag JM , Insel TR , Young LJ . Oxytocin in the medial amygdala is essential for social recognition in the mouse. J Neurosci. 2001;21(20):8278‐8285. 10.1523/JNEUROSCI.21-20-08278.2001. 11588199 PMC6763861

[43] Burkett JP , Andari E , Johnson ZV , Curry DC , de Waal FBM , Young LJ . Oxytocin‐dependent consolation behavior in rodents. Science. 2016;351(6271):375‐378. 10.1126/science.aac4785. 26798013 PMC4737486

[44] Insel TR , Winslow JT , Witt DM . Homologous regulation of brain oxytocin receptors. Endocrinology. 1992;130(5):2602‐2608. 10.1210/endo.130.5.1315251. 1315251

[45] Artymyshyn R , Smith A , Wolfe BB . The use of 3H standards in 125I autoradiography. J Neurosci Methods. 1990;32(3):185‐192. 10.1016/0165-0270(90)90139-7. 2385135

[46] Schneider CA , Rasband WS , Eliceiri KW . NIH image to ImageJ: 25 years of image analysis. Nat Methods. 2012;9(7):671‐675. 10.1038/nmeth.2089. 22930834 PMC5554542

[47] Paxinos G , Franklin KBJ . Paxinos and Franklin's the Mouse Brain in Stereotaxic Coordinates. Cambridge, Massachusetts: Academic Press; 2019.

[48] Ritz C , Baty F , Streibig JC , Gerhard D . Dose‐response analysis using R. Plos One. 2015;10(12):e0146021. 10.1371/journal.pone.0146021. 26717316 PMC4696819

[49] Bates D , Mächler M , Bolker B , Walker S . Fitting linear mixed‐effects models using lme4. J Stat Softw. 2015;67(1):1‐48. 10.18637/jss.v067.i01.

[50] Viviani D , Charlet A , van den Burg E , et al. Oxytocin selectively gates fear responses through distinct outputs from the central amygdala. Science. 2011;333(6038):104‐107. 10.1126/science.1201043. 21719680

[51] Rydén G , Sjöholm I . Half‐life of oxytocin in blood of pregnant and non‐pregnant women. Eur J Endocrinol. 1969;61(3):425‐431. 10.1530/acta.0.0610425. 5820054

[52] Goode TD , Leong K‐C , Goodman J , Maren S , Packard MG . Enhancement of striatum‐dependent memory by conditioned fear is mediated by beta‐adrenergic receptors in the basolateral amygdala. Neurobiol Stress. 2016;3:74‐82. 10.1016/j.ynstr.2016.02.004. 27981180 PMC5146203

[53] Campbell‐Smith EJ , Holmes NM , Lingawi NW , Panayi MC , Westbrook RF . Oxytocin signaling in basolateral and central amygdala nuclei differentially regulates the acquisition, expression, and extinction of context‐conditioned fear in rats. Learn Mem. 2015;22(5):247‐257. 10.1101/lm.036962.114. 25878137 PMC4408769

[54] Oettl L‐L , Ravi N , Schneider M , et al. Oxytocin enhances social recognition by modulating cortical control of early olfactory processing. Neuron. 2016;90(3):609‐621. 10.1016/j.neuron.2016.03.033. 27112498 PMC4860033

[55] Lukas M , Toth I , Veenema AH , Neumann ID . Oxytocin mediates rodent social memory within the lateral septum and the medial amygdala depending on the relevance of the social stimulus: male juvenile versus female adult conspecifics. Psychoneuroendocrinology. 2013;38(6):916‐926. 10.1016/j.psyneuen.2012.09.018. 23102690

[56] Jiang Y , Platt ML . Oxytocin and vasopressin flatten dominance hierarchy and enhance behavioral synchrony in part via anterior cingulate cortex. Sci Rep. 2018;8(1):8201. 10.1038/s41598-018-25607-1. 29844336 PMC5974023

[57] Yamagishi A , Lee J , Sato N . Oxytocin in the anterior cingulate cortex is involved in helping behaviour. Behav Brain Res. 2020;393:112790. 10.1016/j.bbr.2020.112790. 32603799

[58] Raam T , McAvoy KM , Besnard A , Veenema AH , Sahay A . Hippocampal oxytocin receptors are necessary for discrimination of social stimuli. Nat Commun. 2017;8(1):2001. 10.1038/s41467-017-02173-0. 29222469 PMC5722862

[59] Lin Y‐T , Hsieh T‐Y , Tsai T‐C , Chen C‐C , Huang C‐C , Hsu K‐S . Conditional deletion of hippocampal CA2/CA3a oxytocin receptors impairs the persistence of long‐term social recognition memory in mice. J Neurosci. 2018;38(5):1218‐1231. 10.1523/JNEUROSCI.1896-17.2017. 29279308 PMC6596267

[60] Choe HK , Reed MD , Benavidez N , et al. Oxytocin mediates entrainment of sensory stimuli to social cues of opposing valence. Neuron. 2015;87(1):152‐163. 10.1016/j.neuron.2015.06.022. 26139372 PMC4689302

[61] Resendez SL , Namboodiri VMK , Otis JM , et al. Social stimuli induce activation of oxytocin neurons within the paraventricular nucleus of the hypothalamus to promote social behavior in male mice. J Neurosci Off J Soc Neurosci. 2020;40(11):2282‐2295. 10.1523/JNEUROSCI.1515-18.2020. PMC7083279 32024781

[62] Proulx É , Young EJ , Osborne LR , Lambe EK . Enhanced prefrontal serotonin 5‐HT 1A currents in a mouse model of Williams‐Beuren Syndrome with low innate anxiety. J Neurodev Disord. 2010;2(2):99‐108. 10.1007/s11689-010-9044-5. 20585377 PMC2882561

[63] Lew CH , Groeniger KM , Hanson KL , et al. Serotonergic innervation of the amygdala is increased in autism spectrum disorder and decreased in Williams Syndrome. Mol Autism. 2020;11(1):12. 10.1186/s13229-019-0302-4. 32024554 PMC7003328

[64] Campeau S , Davis M . Involvement of the central nucleus and basolateral complex of the amygdala in fear conditioning measured with fear‐potentiated startle in rats trained concurrently with auditory and visual conditioned stimuli. J Neurosci. 1995;15(3):2301‐2311. 10.1523/JNEUROSCI.15-03-02301.1995. 7891168 PMC6578144

[65] Lehmann K , Lesting J , Polascheck D , Teuchert‐Noodt G . Serotonin fibre densities in subcortical areas: differential effects of isolated rearing and methamphetamine. Dev Brain Res. 2003;147(1):143‐152. 10.1016/S0165-3806(03)00130-5. 14741759

[66] Avery SN , Clauss JA , Blackford JU . The human BNST: functional role in anxiety and addiction. Neuropsychopharmacology. 2016;41(1):126‐141. 10.1038/npp.2015.185. 26105138 PMC4677124

[67] Moore RY , Halaris AE , Jones BE . Serotonin neurons of the midbrain raphe: ascending projections. J Comp Neurol. 1978;180(3):417‐438. 10.1002/cne.901800302. 77865

[68] Toth I , Neumann ID , Slattery DA . Central administration of oxytocin receptor ligands affects cued fear extinction in rats and mice in a timepoint‐dependent manner. Psychopharmacology (Berl). 2012;223(2):149‐158. 10.1007/s00213-012-2702-4. 22526533

[69] Gunduz‐Cinar O , Brockway ET , Castillo LI , Pollack GA , Erguven T , Holmes A . Selective sub‐nucleus effects of intra‐amygdala oxytocin on fear extinction. Behav Brain Res. 2020;393:112798. 10.1016/j.bbr.2020.112798. 32653556 PMC7426999

[70] Chailangkarn T , Trujillo CA , Freitas BC , et al. A human neurodevelopmental model for Williams Syndrome. Nature. 2016;536(7616):338‐343. 10.1038/nature19067. 27509850 PMC4995142

[71] Menon R , Grund T , Zoicas I , et al. Oxytocin signaling in the lateral septum prevents social fear during lactation. Curr Biol. 2018;28(7):1066‐1078.e6. 10.1016/j.cub.2018.02.044. 29551417

[72] Guzmán YF , Tronson NC , Jovasevic V , et al. Fear‐enhancing effects of septal oxytocin receptors. Nat Neurosci. 2013;16(9):1185‐1187. 10.1038/nn.3465. 23872596 PMC3758455

[73] El‐Ghundi M , O'Dowd BF , George SR . Prolonged fear responses in mice lacking dopamine D1 receptor. Brain Res. 2001;892(1):86‐93. 10.1016/S0006-8993(00)03234-0. 11172752

[74] Darvas M , Fadok JP , Palmiter RD . Requirement of dopamine signaling in the amygdala and striatum for learning and maintenance of a conditioned avoidance response. Learn Mem. 2011;18(3):136‐143. 10.1101/lm.2041211. 21325435 PMC3056517

[75] Gainetdinov RR , Mohn AR , Bohn LM , Caron MG . Glutamatergic modulation of hyperactivity in mice lacking the dopamine transporter. Proc Natl Acad Sci. 2001;98(20):11047‐11054. 10.1073/pnas.191353298. 11572967 PMC58681

